# Review On the Effects Curcumin on Tumors of the Reproductive System: Curcumin and Reproductive Tumors

**DOI:** 10.31661/gmj.v10i0.2178

**Published:** 2022-07-07

**Authors:** Moradi Zahra, Tohidian Mostafa, Hekmatnia Yasaman, Dalili Amin, Sadeghi Mostafa, Neshat Seyed Sina, Jamalnia Sheida, Reihani Elnaz

**Affiliations:** ^1^ Shahid Beheshti University of Medical Sciences, Tehran, Iran; ^2^ Islamic Azad University of Medical Science, Sari, Mazandaran, Iran; ^3^ Surgical Oncology Research Center, Mashhad University of Medical Science, Mashhad, Iran; ^4^ Department of Operating Room, Montaserieh Dialysis and Transplant Center, Mashhad University of Medical Sciences, Mashhad, Iran; ^5^ Student Research Committee, School of Medicine, Isfahan University of Medical Sciences, Isfahan, Iran; ^6^ Shiraz University of Medical Sciences, Shiraz, Iran; ^7^ Molecular cell biology, Hakim Sabzevari university, Sabzevar, Iran

**Keywords:** Curcumin, Reproduction, Cancer, Tumor, Prostate, Ovary, Breast

## Abstract

Curcumin, a polyphenolic derivative of Curcuma longa rhizome, has numerous beneficial effects, including antibacterial, anti-inflammatory, antiviral, antioxidant, antifungal, anti-ischemic, anti-cancer, hypoglycemic, nephroprotective, antirheumatic, hepato-protective, and antimutagenic. Curcumin has indicated the capability to exert anti-cancer activity by multifunctional mechanisms, such as induction of apoptosis, inhibition of cancer cell proliferation, cell cycle regulation, chemotherapeutic intestinal absorption, and modification of several cancer cell types signaling pathways. Several studies have shown that curcumin may have protective effects against tumors of the reproductive system. Reproductive system cancers may cause many undesirable physical and, especially, mental disorders. Infertility and its mental consequences, sexual problems, chemotherapy and surgery-related adverse effects, substantial economic burden, and death are the most common complications regarding the cancers of the reproductive system. By modulating several reproductive cancer hallmarks such as signaling pathways, multiple drug resistance, cancer cell growth and proliferation, tumor angiogenesis, and transcription factors, curcumin could be used as a safe, non-toxic, cheap, and easily accessible drug for treating different types of reproductive cancers.

## Introduction

Traditional medicine and medicinal plants came into focus in the recent decade as a result of the credence that herbal products are healthier, having fewer adverse and toxic effects than synthetic drugs [1]. The usage of Curcumin, the main constituent of Curcuma longa with various medicinal properties, was traditionally common in several Asian countries [2]. It is mainly used as cosmetics, drugs, spice, and coloring agents in various foods [3]. Various disorders have been reported to improve by beneficial effects of curcumin, including metabolic syndrome, osteoporosis, cancer, hypertriglyceridemia, depression, anxiety, osteoarthritis, and non-alcoholic fatty liver disease [4,5,6,7,8,9]. Curcumin may act as an anti-infectious, anticarcinogenic, antifungal, anti-inflammatory, antiparasitic, antiviral, antioxidant, and antimutagenic agent [10,11,12]. The main curcuminoid of C. longa is curcumin (77%), followed by desmethoxycurcumin (17%) and bisdemethoxycurcumin (3%) [13]. Due to low absorption in the intestines, curcumin rapidly disappears from circulation [14]. In the liver, curcumin is metabolized by glucuronidation and sulfation and then excreted in the feces and urine [15,16]. Curcumin is generally safe, and even at high doses, it is not toxic to animals and humans [17,18]. However, some studies have reported adverse effects, such as nausea, diarrhea, ulcers, rash, and yellow stool [19,20]. Several studies have reported the effects of curcumin on the sexual glands, ovaries, breasts, testis, and endometria that could be attributed to its anti-inflammation [21], anticancer [22], anti-inflammatory [21], antioxidant [23], anti-apoptotic [24] properties. Therefore, the present review aimed to investigate the impacts of curcumin on prostate, testis, breast, ovarian, and endometrial cancers.

## Effects of Curcumin on Prostate Cancer

Prostate cancer is considered the second cause of death among men in Western countries [25]. Chronic inflammation is the leading risk fac-tor for prostate cancer development. Metas-tasis of prostate cancer cells is also triggered as a result of chronic inflammation [25]. Sev-eral studies have suggested that the main an-ti-cancer action of curcumin is inhibiting the nuclear factor kappa B (NFκB), which plays a significant role in both inflammatory and apoptosis pathways [26]. By inhibiting kap-paB kinase [27,28], curcumin inhibits NFκB translocation and the resulting inflammato-ry response. Killian et al. [25] reported that curcumin disrupts the CXCL1/-2 and NFκB pro-inflammatory signaling pathways, thus inhibiting metastasis of prostate cancer cells. Therefore, curcumin administration can sup-press metastasis of cancer cells in addition to inhibiting prostate cancer development. Androgen receptors are crucial for the devel-opment and progression of prostate cancer due to their necessity for prostate function and growth [29,30,31]. Curcumin can alter sev-eral signaling pathways of the androgens that play a role in the growth and development of prostate cancer cells [32,33,34,35,36,37]. Furthermore, curcumin can downregulate the production of androgens and their receptors [38]. By down-regulating androgen receptor expression, cur-cumin may prevent the androgen-mediated expression of prostate-specific antigen [38]. Some studies have reported that the apoptotic effects of curcumin on prostate cancer cells are due to its inhibitory effects on androgen synthesis and androgen receptor expression [39]. Since androgens are necessary for the growth and development of prostate cancer cells, inhibiting their signaling results in the stimulation of apoptosis. Some other stud-ies have indicated that apoptosis inhibition in prostate cancer cell lines is due to the re-duction of anti-apoptotic proteins expression by curcumin, such as Bcl-2 and Bcl-XL [29,40]. It is well known that chronic inflamma-tion could lead to the metastasis of prostate cancer cells by regulating the prometastatic and pro-inflammatory feedback loop between CXCL1/-2 and NFκB. By disrupting this pos-itive feedback loop and NFκB signaling path-way, curcumin can prevent prostate cancer cell metastasis[25]. In conclusion, curcumin exerts its protective effects on prostate cancer by inhibiting the growth, proliferation, and metastasis of prostate cancer cells through modulating several inflammatory and oxida-tive signaling pathways. Investigating its ben-eficial effects along with/in combination with other anti-prostate cancer drugs is of high importance to introduce new therapeutic ap-proaches, especially based on herbal drugs for the treatment of this type of cancer with the lowest adverse effects.

## Curcumin Effects on Breast Cancer

The most prevalent cancer in women, especially in developed countries, is breast cancer [41]. Based on the expression of biochemical markers, breast cancer is categorized into several types, including estrogen receptor and progesterone receptor weak positive (luminal B), estrogen receptor and progesterone recep-tor strong positive (luminal A), Her2 positive, and triple-negative [21]. Approximately 70% of breast cancers are estrogen receptor-posi-tive, showing positive treatment results after anti-estrogen treatment and hormone thera-py [42,43]. Triple-negative breast cancer is a very aggressive and hard-to-treat disease due to the lack of receptors to serve as a ther-apeutic target [44]. Upregulation of androgen receptors is reported in triple-negative breast cancer, and it seems that the inhibition of these receptors’ expression or action could be considered as a therapeutic approach in the treatment of this type of cancer. Radiotherapy, chemotherapy, and surgery are the main ther-apeutic strategies for treating breast cancer [45]. Due to the drug resistance, poor patient response, and high possibility of relapse [46], many researchers are trying to introduce nov-el therapeutic strategies to treat different types of breast cancer. There are many studies regarding the pro-tective effects of curcumin on breast cancer. The main mechanisms by which curcumin inhibits the growth and development of this cancer include modifying cell signaling path-ways and molecules (such as phosphatidyli-nositol-3-kinase, Ras, mammalian target of rapamycin, Wnt/β-catenin, and protein kinase B), induction of p53-related apoptosis, and arresting cell cycle, preventing angiogene-sis, and tumor growth, and inhibition of some transcription factors [46]. Additionally, cur-cumin exerts some of its anti-tumor effects by targeting the estrogen receptor signaling path-way and other signaling pathways. Curcumin inhibits the proliferation of breast cancer cells by significantly downregulating estrogen re-ceptor α, estrogen receptor β, and leptin in a dose-dependent manner [47]. Administrated 20 to 80 μM of curcumin leads to estrogen re-ceptor α and p53 protein expression markedly reduced [48]. Estrogen receptor-positive cell lines such as MCF7 and BT474 have a higher half-maximal inhibitory concentration (IC50) for curcumin than estrogen receptor-negative cell lines (e.g., MBA-MB-231 and SKBR3) [49,50]. It shows that curcumin may ex-ert some of its protective effects by other mechanisms and pathways. In most breast cancer cell lines, curcumin stimulates apop-tosis by changing cell membrane potential [51]. Several studies have reported that cur-cumin can upregulate caspase 3 and caspase 9 expression and induce the mitochondrial release of cytochrome C [52,53]. This may explain mitochondrial-dependent apoptotic pathway induction by curcumin. Other cur-cumin-dependent apoptotic pathways include the downregulation of Bcl-XL and Bcl-2 ex-pression and the upregulation of Bax and Bad anti-apoptotic proteins [52]. Angiogenesis is another vital factor for tumors and cancer pro-gression [54]. To provide nutrients and blood flow, tumor cells produce some pro-angio-genic molecules such as vascular endothelial growth factor (VEGF) and basic fibroblast growth factor [54,55]. Findings suggest that breast cancer growth and angiogenesis are inhibited by curcumin administration [46]. By downregulating VEGF isomers, curcum-in can efficiently prevent tumor angiogenesis in breast cancer [55]. In conclusion, the main mechanisms by which curcumin exerts its anti-cancer effects against breast cancer are promoting apoptosis of breast cancer cells, presentation of angiogenesis, preventing cell growth signaling pathways, and inhibiting es-trogen receptor signaling pathways. Curcum-in can be used as a novel treatment along with other current therapeutics to prevent, and treat breast cancer.

## Effects of Curcumin on Ovarian Cancer

The most lethal cancer of the reproductive system is ovarian cancer [56]. It has a high mortality rate due to the development of che-moresistance, late diagnosis, and a lack of effective treatment strategies [57,58]. Low treatment efficiency is due to the aggressive-ness of the disease, mark tumor heterogene-ity, and early metastasis [59,60]. In general, ovarian cancer is classified into three types; epithelial (the most common), sex-cord-stro-mal, and germ cell [61]. Many modifiable and non-modifiable risk factors are related to ovarian cancer. The modifiable risk factors in-clude smoking, dietary factors, and hormon-al replacement therapy. Non-modifiable risk factors include family history, race, endome-triosis lynch syndrome, BRCA1 and BRCA2 mutation carrier. Ovarian cancer treatment is generally based on surgery, chemotherapeutic agents, intraperitoneal chemotherapy, and vi-tamin D supplementation [61]. Several studies have indicated the protective effects of curcumin and its derivatives against ovarian cancer. Some suggest that curcumin exerts its anti-tumor function by activating the pro-apoptosis proteins and inhibiting the anti-apoptosis proteins [62]. In the Ho-8910 ovarian cancer cell line, curcumin marked-ly prevented the growth and proliferation of these cells by downregulating Bcl-XL and Bcl-2, and upregulating Bax and p53 proteins [62]. This shows that curcumin could induce apoptosis in ovarian cancer cells. In cispla-tin-resistant ovarian cancer cells, curcumin inhibited cell proliferation by G2/M arrest, superoxide generation, and apoptosis [63]. In cisplatin-sensitive and resistant ovarian cancer cells, superoxide production was in-creased. Curcumin arrested the cell cycle in the G2/M phase by inducing p53 protein phos-phorylation and caspase 3 activations. Akt phosphorylation was also inhibited after cur-cumin administration, and cell proliferation stopped [63]. Some other studies have sug-gested that curcumin may regulate the SFRP5 gene, which is important in the Wnt/β-catenin signaling [64]. In SKOV3 ovarian cancer cell lines, curcumin inhibited colony formation and cell migration by epithelial-mesenchymal transition [64].

Furthermore, ovarian cancer drug resistance could be ameliorated by curcumin. The sen-sitivity of ovarian cancer cells to cisplatin has been shown to increase by FA/BRCA path-way inhibition in a dose-dependent manner [65]. Multiple drug resistance (MDR) is a mechanism by which cancer cells overcome anti-cancer drugs. A primary mechanism for MDR is effluxing drugs by cancer cells so that they can escape from the toxic effects of anti-cancer drugs [66]. In animals with MDR ovarian cancer tumors, curcumin adminis-tration significantly decreased tumor growth [67]. Additionally, curcumin significantly in-creased intestinal absorption of chemothera-peutic drugs and resulted in IC50 reduction in SKOV3 ovarian cancer cell line [68]. In con-clusion, curcumin shows its improving effects on ovarian cancer by several main pathways, including induction of apoptosis by modulat-ing anti-apoptotic and pro-apoptotic proteins, inhibition of cancer cell proliferation by the cell-cycle arrest in the G2/M phase, increas-ing intestinal absorption of chemotherapeutic drugs, and inhibition of MDR.

## Conclusion

The findings from previous studies have shown that curcumin exerts its anti-cancer ef-fects by targeting several signaling pathways and molecules. By modulating several repro-ductive cancer hallmarks such as signaling pathways, multiple drug resistance, cancer cell growth and proliferation, tumor angio-genesis, and transcription factors, curcum-in could be used as a safe, non-toxic, cheap, and easily accessible drug for treating differ-ent types of reproductive cancers. However, the poor availability of curcumin restricts its clinical use, and specific novel approaches are required to solve this problem. Many in-vestigations are in progress on new delivery systems, including encapsulating curcumin into liposomes or lipid micelles, nanotech-nology-based formulations, conjugation with antibodies or other specific ligands, and the use of curcumin analogs, which consist of the same properties but better bioavailability to overcome the limitations regarding curcumin poor bioavailability.

## Conflicts of Interest

None.
